# A Step Too Far? Public Perceptions on Navigating the Introduction of Robot Technologies into the Human–Animal Relationship in Agriculture

**DOI:** 10.3390/ani16182830

**Published:** 2026-09-09

**Authors:** Cheryl Travers, Robert Sparrow, Angie Sassano, Megan Frances Moss, Chris Degeling

**Affiliations:** 1 Australian Centre for Health Engagement, Evidence and Values (ACHEEV), University of Wollongong, Wollongong 2522, Australia; 2 Department of Philosophy, Monash University, Melbourne 3800, Australiaangie.sassano@monash.edu (A.S.); megan.moss@monash.edu (M.F.M.)

**Keywords:** animal agriculture, animal welfare, ethics, human–animal relationship, robotics

## Abstract

Robots are being developed to perform the day-to-day operational tasks of animal agriculture, affecting the way in which humans and farm animals interact. There is the prospect of automation increasing to the point where humans would hardly set foot on farms, which, aside from the consumption of animal-based products, could entirely disconnect humans and farmed animals from each other. Yet the literature on public attitudes towards the introduction of agricultural robots into the human–animal relationship is sparse. Here, we consider the public acceptability of introducing robots into the human–animal relationship in agriculture. Between 2023 and 2025, we conducted 12 dialogue groups and two community juries with members of the Australian public on the social and ethical impacts of automation in agriculture. We draw on insights from these empirical studies to highlight the underlying values that inform different attitudes towards robots taking on tasks previously performed by humans in animal farming. Across our empirical work, we consistently found three key factors influencing the willingness of members of the public to accept agricultural robots into the human–animal relationship: moral responsibility for farm animals; human intuition and empathy; and a natural life as a condition of animal welfare. Our data suggested that the removal of humans from all contact with farmed animals by adopting highly roboticized farming will likely be rejected by the Australian public as “a step too far”, largely due to its moral and social implications. We identify a set of conditions that are likely to be important to public acceptability of the use of robots in animal agriculture.

## Simple Summary

With robots increasingly designed to perform the tasks of animal agriculture, humans may become entirely disconnected from farmed animals. Our research studies with members of the Australian public indicate that people’s willingness to accept agricultural robots into the human–animal relationship will depend on factors such as human responsibility for and oversight of farm animals, the importance of human intuition and empathy in animal care, and the provision of a natural life as a condition of farm animal welfare. The findings highlight that important ethical and policy questions will arise should agricultural robots impact the human–animal relationship. We identify a set of conditions that are likely important to public acceptability of robotics in animal agriculture.

## 1. Introduction

Robots are being developed to perform the day-to-day operational tasks of animal agriculture. Increasingly, modern robots use Artificial Intelligence (AI) to allow them to operate as “independent functioning intelligent systems”, able to perceive their environment and respond flexibly, and capable of something akin to “’intelligent’, ‘rational’ and ‘autonomous’” behaviour [1]. Should they replace humans on farms, the integration of such robots into animal agriculture would inevitably alter the nature of the relationship between humans and farm animals. There is a prospect of this technology being developed to the point where, in some food production systems, there is minimal or no human presence on the farm [2,3,4]. Inserting robots into the human–nonhuman animal (hereafter human-animal) relationship raises ethical and animal welfare concerns. One concern is that these technologies risk alienating humans from livestock, thereby eroding embodied contact and familiarity between animals and farmers [5].

Although societal attitudes are known to influence technological transformation in agriculture [6], studies on public perspectives regarding the use of robotics in animal agriculture are limited. To help fill this research gap, this paper explores public perceptions about introducing robots into animal agriculture; specifically, it focuses what members of the Australian public think about the implications of the use of this technology for the human–animal relationship and when/if it might be acceptable.

Public perception of robotics in animal agriculture is important, as consumer purchasing habits directly determine the success of farm animal value chains [7]. There is growing evidence that public acceptance of livestock production now centres on animal sentience, welfare, public health risks and environmental sustainability, rather than traditional food security arguments. Public debates also have the power to shape future policy [8], and the use of these technologies should have social license. Yet, existing empirical research on agricultural robotics largely neglects the question of—and the conditions for—social acceptability regarding these technologies in animal agriculture [7,8], including the implications for the human–animal relationship.

In our previously published work, we have drawn on data gathered from 12 dialogue groups and two community juries as part of a larger project conducted to understand public perceptions on the ethics of AI and robotics in agriculture. Specifically, Sassano et al. 2025 [9] presented the findings from dialogue groups which were conducted across both metropolitan and rural Australia, and investigated public perceptions regarding the social and ethical impacts of AI and robotics in agriculture. By mapping participant sentiments toward hypothetical scenarios on automation and the ‘farmerless farm’, this study reveals how the public ethically assesses the value of these technologies. The study found that many members of the public view AI and robot technologies with scepticism. Participants questioned how the food system should be structured, invoking agrarian imaginaries to uphold the moral value of farming. Participants’ concerns about animal welfare focused on intensive industrialised farming—particularly poultry factories—and their struggle to balance animal welfare against the human benefits of enhanced biosecurity measures.

A subsequent paper by Sassano et al. (2026) [10] reported the findings of two community juries which deliberated on the conditions, if any, under which the full automation of food and fibre production should occur. This paper described the deliberative process undertaken to better understand how different imaginaries around Australian agriculture influence the publics’ willingness to accept ‘farmerless farms’. Participants’ views on animal welfare were explored by means of asking them to consider the possibility of automating animal agriculture. Most members of both juries expressed concern regarding fully automated animal agriculture, because they highly valued human moral agency in interactions with sentient animals, including during the slaughter process.

Although these previous papers highlighted public concerns regarding the welfare of farmed animals (primarily poultry), neither examined the introduction of robotic technologies in relation to the human–animal relationship. Furthermore, the original research project which generated these findings, was not designed to investigate this critical issue; the findings emerged directly from the participant data.

The current paper is a companion article to these studies and expands our analyses of the values that underpin and inform public views and intuitions about robotics in animal agriculture. Our paper is novel in exploring the under-researched topic of public perceptions on robotic technologies and the human–animal relationship in agriculture. Specifically, our focus is on participant views regarding the impact and acceptability of robots on the human–animal relationship in agriculture. By drawing on insights from our multiple structured engagements with members of the Australian public, we identify a set of conditions that are likely to be important when it comes to public acceptability of the use of robots in animal agriculture. We found three key factors that influence the willingness of members of the public to accept agricultural robots into the human–animal relationship: moral responsibility for farm animals; human intuition and empathy; and a natural life as a condition of animal welfare.

In the background section, we briefly describe current conceptualisations of the human–animal relationship in agriculture and the importance of this relationship to animal welfare, and summarise some of the findings in the literature regarding the risks and benefits of the use of robots. We then present our methods and the data analyses relevant to this companion article. We go on to share our findings from participant responses to issues surrounding the human–animal relationship and the introduction of robotics to animal agriculture. We discuss the academic literature in relation to the implications of our findings and conclude with some observations on navigating the introduction of agricultural robots into human–animal relationships.

## 2. Background

The food industry commonly uses the guiding principles of the Five Freedoms or, more recently, the Five Domains Model for assessing animal welfare [11]. With these principles in mind, the requirements for adequate animal welfare in agriculture can be broadly grouped under the objectives of: ensuring basic good health and functioning; minimising unpleasant affective states (e.g., pain fear, frustration and anxiety) and allowing animals normal pleasures; and providing them the ability to lead reasonably natural lives [12,13]. A growing scholarly discourse advocates moving beyond these basic requirements toward a ‘life worth living’ for farmed animals, by minimising negative experiences while providing greater opportunity for positive ones, and allowing them to express their harmless natural preferences, needs and wants [14,15].

Today, animal welfare science balances traditional metrics of suffering and biological functioning with affective states and natural living [16,17]. While natural living is a criterion to assess how we care for animals, it remains an ambiguous concept. [18]. In this paper, we adopt the term natural living to refer generally to the freedom for nonhuman animals to express their natural behaviours. Different criteria can be combined to assess natural behaviours; for instance, evaluating “what animals want” helps identify which behaviours are important to their welfare [12,13]. However, despite being commonly utilised to assess farm animal interventions and gauge public perceptions of animal welfare, natural behaviour is a complex concept that remains challenging to apply objectively [18].

The human–animal relationship is an important factor in the welfare of farmed animals. Erin Ryan and colleagues define “relationship” as “any intertwining or interdependency of two lives, regardless of the duration and degree of direct physical contact” [19]. In considering the escalating uptake of robotics in livestock agriculture, this definition allows us to reflect on two fundamental ways the human–animal relationship may be framed. In agriculture, the human–animal relationship largely develops between the farmer or stockperson and farm animals, by “the mutual perception, which develops and expresses itself in their mutual behaviour” [20]. The quality of this “subjective relationship” is shaped by the nature of human interaction, typically qualified as good (e.g., friendly and trustworthy), neutral, or negative (e.g., featuring punishments) [20,21,22,23]. There also exists a socially distant, “objective” relationship between consumers and farm animals, characterised by little or no direct contact. Yet it is a powerful relationship, in that consumers can drive change in farming practices and, as such, wield influence over the lives of farmed animals [24]. The introduction of robots would likely affect both sorts of human–animal relationships. Here, we largely focus on the impact of robotics on the subjective relationship, which was the primary concern of our participants.

Human–animal relationships on the farm differ across production systems and species. For instance, dairy cow farmers tend to develop close relations with their cows, while poultry farmers are more distant from the animals in poultry production [22]. Despite the differences in the degree of relational closeness between farmers and their animals, and improved farming technologies, some scholars consider a healthy human–animal relationship as fundamental to animal welfare in agricultural systems [23]. Studies have demonstrated that a good human–animal relationship has a positive effect on animal health, stress, resilience and performance, which in turn can improve productivity and quality of produce [22,23,25,26]. George and Bolt (2023) emphasise that positive attitudes among stockpeople are essential for safe farm practices, while also enhancing worker job satisfaction and self-esteem [25].

Most research on robotics in animal agriculture focuses on dairy cow farms and automated milking systems, which have transformed routine herd milking by allowing individual cows to choose when they are milked [27]. Some farmers affirm that robotic milking reduces the physical demands and constraints placed on themselves and cows. Hence, cows have more freedom and autonomy, and farmers have the opportunity to more fully participate in their family and social lives [5].

However, as automation alters the way humans and animals interact, robotic technologies raise the possibilities of risks and harms [15]. For example, the successful operation of automated milking systems relies on the interpretation of data collected by AI and robotics rather than direct human–animal contact [6]. In turn, farmers risk losing their affinity for their dairy cows, transforming the animals from sentient beings into quantifiable objects [5,8,28]. For animals subject to intensive production, the datafication of robotic systems reduces their life to mere “elements of a system” [28]. Farm animals typically fear humans unless they are neutrally or positively habituated to their presence [20,21]. Should robots perform everyday tasks, farm animals would be less habituated to humans, potentially eliciting panic during inevitable human interactions and risking injury to both handlers and animals [20,29].

Advanced robotic technologies present unique challenges in poultry production, as welfare management still operates at a flock level, making it difficult to ensure the wellbeing of individual birds [30]. Poultry birds are, nonetheless, sentient beings, “having the capacity, the level of awareness and cognitive ability, necessary to have feelings” [31]. Domesticated chickens use logical reasoning, complex communication, and form dynamic social networks [32]. Yet, they are often considered to possess low levels of intelligence, making it easy for people to commodify them [32] and neglect their welfare for the sake of productivity.

Against this background, while wealthy societies are, in the main, disconnected from the realities of animal production systems [33], empirical studies highlight consumer interest and concern for animal welfare and the human–animal relationship in farming [7,34,35,36]. Social acceptance of robots in animal agriculture will, therefore, depend on what the public thinks about the impacts on animal welfare and human–animal relationships, as well as, perhaps, the efficiency and productivity of animal farms—metrics that are often the priorities of industry.

## 3. Methods

Between 2023 and 2025, we conducted research on the social and ethical impacts of AI and robots in agriculture, focusing on the attitudes, values and moral intuitions that inform members of the public’s acceptance or rejection of these technologies. To do so, a research team including all of the authors convened 12 dialogue groups (*n* = 67) and two community juries (*n* = 23), conducted across metropolitan and regional Australia.

Participants for both studies were recruited by a professional recruitment service—the Taverner Research Group, using selection criteria based on socio-economic indicators (e.g., gender, age, occupation, postcode, and education) to ensure fair representation of the communities involved in each study. The dialogue group sample was fairly balanced by gender, with a slightly higher proportion of women (*n* = 38, 57%) than men (*n* = 29, 43%). The majority of dialogue group participants had no connection to agriculture, though some rural individuals had family ties to the sector or worked in related industries. Metropolitan groups comprised individual consumers [9]. The community jury groups were also fairly balanced by gender. In contrast to the Mildura community jury cohort—where all 11 participants had lived or worked in a community reliant on agriculture—only two Melbourne participants shared this experience [10].

For interested readers, the complete methodology, including research materials, is reported in Sassano et al. (2025) for the dialogue groups, and Sassano et al. (2026) for the community jury research. The current paper draws on previously unreported data from these deliberative fora in order to identify a set of conditions likely important to the public acceptability of introducing robots into the human–animal relationship in farming. In the following section, we provide a concise description of methods and data analysis for the dialogue groups and community juries, with relevance to this companion article.

### 3.1. Dialogue Groups

In dialogue groups, a facilitator presents hypothetical scenarios to participants to better understand their views, values, and moral insights on specific topics [37]. Dialogue groups begin with a brief explanation of the topic, followed by a presentation of a hypothetical scenario, then a facilitated discussion around what participants think should be done in the scenario and the factors that might influence their decisions. In our research, we asked the dialogue groups for their moral intuitions and judgements on AI and robot technologies in agriculture. A facilitator presented two hypothetical scenarios covering horticulture and animal agriculture—specifically poultry production. During these sessions, a facilitator introduced changes to the scenarios to clarify the decisions expressed by participants.

Our 12 dialogue groups (DGs) were conducted between August and December 2023: six groups held across metropolitan areas of Melbourne (Victoria), Sydney (New South Wales), and Brisbane (Queensland), and six involving participants from regional farming areas in these States. Ethics approval was provided by the Deakin University Human Research Ethics Committee (HAE-23–068). Once Taverner provided a list of individuals willing to participate, a member of the research team emailed each individual a Participant Information Statement and a link to an online Qualtrics consent form. Follow-up contact was then made by phone to answer participant questions, confirm study details, and offer any support required. The facilitator-led dialogue groups were held via Zoom for 90 min, with each group comprising up to seven participants. These sessions were audio-recorded and professionally transcribed.

For the larger research study, the results of which are reported in Sassano et al. (2025), we used the Framework Method to analyse dialogue group data [38]. Three team researchers all read the first three transcripts to develop an initial coding framework. The texts were then hand-coded and categorised using deductive and inductive codes. The remaining transcripts were divided between two coders, while the third coder conducted an audit to ensure consistency in data interpretation. The process was iterative, with weekly discussions between the three coders and fortnightly sessions with the broader team. The coded data was charted on a Microsoft 365 Excel spreadsheet. The analysis highlighted the potential impact of AI and robotics on animal welfare as a strong, emergent theme. This finding sparked team discussions regarding potential participant concerns about the human–animal relationship and prompted us to investigate the matter further: we report the results of that investigation in this paper.

### 3.2. Community Juries

A community jury aims to elicit the perspectives and preferences of groups of people who are provided with education about, and time to discuss and address, a specific topic or problem [39]. Ethics approval was provided by the University of Wollongong Human Research Ethics Committee (2024/199). Typically, a community jury is conducted over 1–3 days, during which 12–15 members of the public (i.e., jurors) are given the following: in-depth and balanced information by experts on the topic under consideration; opportunity to “cross-examine” the experts and engage in reasoned dialogue—helping jurors broaden their views through considering the insights of others; and adequate time for jury deliberation and recommendations [40,41].

In 2025, we conducted two in-person community juries to explore the group’s perspectives on the conditions (if any) under which complete automation of farms and agricultural systems is acceptable. We held the first jury (CJ1, *n* = 12) in an inner suburb of Melbourne (capital city of Victoria), largely involving consumers, with the second jury (CJ2, *n* = 11) held in Mildura, a regional city in Victoria with a local economy reliant on agriculture.

Prior to the community juries, participants received a document detailing the question they were being asked to consider, and basic factual information on five relevant topic areas to be covered by five “expert witnesses”. The expert witnesses were selected according to their institutional roles and expertise in their topic. An orientation session held on Day 1 introduced the process and the questions for consideration, and obtained participant written consent. The jury then viewed five video presentations, pre-recorded by the expert witnesses, to ensure consistency in the provision of information to both juries. These presentations ran for 15–20 min, followed by a facilitated question-and-answer session, allowing jurors to clarify or question the evidence and opinions presented. Day 2 saw jurors reflect on, discuss, and debate the evidence, aided by a researcher facilitator. The jurors then deliberated without facilitators present to reach a verdict on the question posed. They reported their verdicts, reasoning and any dissenting views in a facilitated feedback session. All jury deliberations and expert question-and-answer sessions were audio recorded and transcribed.

The main outcomes were the jury verdicts and the reasons for their decisions. The review process for the larger study was descriptive in order to confirm and verify the reasons and rationales provided by jurors during the final facilitated feedback session [10].

### 3.3. The Current Study

In summary, the community juries and dialogue groups are different deliberative formats. They also use different methods for data analysis. The dialogue groups used hypothetical scenarios and Framework Analysis, with group discussions (and transcripts thereof) serving the unit of analysis. In contrast, the community juries incorporated expert evidence and extended deliberation, using both the deliberative groups (the two juries) and their verdicts as the units of analysis.

The analysis of, and results from, both sets of data informed the current study. Through repeated readings and conversations with team members, Author 1 identified further codes in the transcripts of the discussions in the dialogue groups related to animal welfare and the human–animal relationship and robots. An informal search using keywords that were identified from these codes (animal welfare; sentience; nature/natural; connectedness; responsibility; accountability; moral; and, duty) was performed on the transcripts of the discussions in the citizens’ juries to identify further contexts in which participants discussed these matters. Repeated readings of the community jury transcripts also helped identify similarities and differences in themes, focusing on automation in animal agriculture and, more broadly, on agriculture as a whole. In addition, Author 1 repeatedly read all community jury transcripts, noting keywords identified during the dialogue group data analysis—and sought the other authors’ input during regular team meetings.

## 4. Findings

In this section, we describe participants intuitions and expressed values when they considered the introduction of robots into the human–animal relationship in farming during these empirical processes. Three different themes emerged from our analyses: sentience, moral duty and responsibility; human intuition and empathy; and that farm animals should enjoy a natural life. Because the dialogue groups generated more discussion on the themes than the community jury deliberations, most of the quotations are drawn from these sessions. However, as Table 1 shows, community jurors also raised and supported these three broad themes.

### 4.1. Sentience, Moral Duty and Responsibility

Most of our participants—both in the dialogue and community jury groups—empathised with, and felt compassion for, animals that they perceived to be sentient beings like humans, capable of suffering [42]. Participants acknowledged animal sentience when discussing how the animals’ “feel” and their ability to experience pain, fear and distress, and happiness. Accordingly, some participants indicated that we have a duty of care for farm animals and should acknowledge their “stress and their lifestyle” (DG3) because when “their needs are met, they are happy” (DG3).

As mentioned, the inclusion of robots in animal agriculture may seriously limit interaction with, and thus visibility of, farm animals, such that humans would not have the opportunity to appreciate farm animal sentience. Dialogue group participants generally construed moral responsibility in these circumstances as a need for human supervision or oversight of robot interactions with sentient animal beings, with one noting

*“… it’s [the animal] a living thing, and there’s cognition there…that’s where a line draws. Like you need to have some sort of oversight with humans…”*

(DG6)

Many others raised human responsibility and accountability for animal welfare should animal agriculture become roboticized, such that

*“… a robot isn’t accountable for anything. So where is the guarantee that animals and sentient beings are going to be treated in a humane manner?”*

(DG1).

One participant contended that the removal of humans from farming would eliminate “human intellect, human empathy”, and that this, in turn, would constitute “removing accountability” (DG1).

Many community jurors also highlighted the importance of human responsibility. When a facilitator suggested that chickens might not care if they are tended by a human or robot, one community juror captured the broad sentiment, “… yes, yes, but we might care. Someone needs [to] take responsibility for the things that happen” (CJ2). Some community jurors even argued that our moral obligations to animals extends to the slaughterhouse, with one participant stating that we “owe a duty of care to individual animals even if what you’re doing is killing them” (CJ1). Indeed, they agreed that humans should retain a sense of moral discomfort in the slaughter process, “for the sake of what makes us human” (CJ1). In effect, our discomfort with the slaughter process was considered an important factor in farm animal welfare.

### 4.2. Human Intuition and Empathy

Participants highlighted the importance of intuition, care and empathy, when it came to the proper treatment of animals. Farmers, they suggested, have an intuitive understanding of the experience of animals—described by some dialogue group and community jury participants as the “human touch”—that cannot be replicated by robots. As one dialogue group participant suggested

*“… [the whole] ethos of the human touch, so to speak, is we have the brain. So no one can swap that out from us. It’s not electronic. Yes, robots will have a brain, but you can’t convey the feeling of empathy and the feelings of like true emotion because they’re not born with it… It’s programmed into them…”*

(DG7).

A medically trained participant highlighted the value of care technologies in physician–patient–robot relations, emphasising the need for human intuition and responsiveness in decision-making:

*“… if you think about those really high stress decisions, where a lot of human error can be made just due to emotions … I think that AI is …. really useful …. However, there should always be a doctor behind the decisions … you have to think, who are the patients that we are basing the generation of this data off of”*

(DG8).

Some dialogue group participants rejected the use of robots in animal farming by emphasising our inherent interdependency with nature, which, they thought, must be preserved. They supported the notion that humans and animals share a subjective experience of life, with one participant claiming, “… all animals are actually linked. It’s in the biology of the world” (DG3).

However, some other participants acknowledged that the human–animal relationship is not always good for either humans or animals. In raising the issue of violence towards animals, one participant with experience in farm work told us

*“… I also know that a lot of chickens get mistreated by people. They get shaken… If a chicken pecks at them, they kick, they hurt the chicken. A robot won’t do that, they won’t get emotional towards the chicken”*

(DG3).

A few dialogue group participants insisted that robots might be capable of providing appropriate care to farm animals, with one suggesting

*“… you can simulate the fact of having a human in terms of machines…You have music…your environment…climate control. There’s other ways to have that same emotional effect on an animal”*

(DG6).

Where most participants agreed—across dialogue and community jury groups— was that, to be acceptable, the use of robots should improve the welfare of sentient animals, whether by offering good “care” understood as a standard procedure, or by minimising the opportunity for intentional human cruelty.

### 4.3. Farm Animals Should Enjoy a Natural Life

Some dialogue group participants advocated methods of agriculture which allow animals to express natural behaviours and facilitate positive human–animal relationships. For instance, one participant drew upon an idea of the farmer talking to their animals, noting

*“… when you praise anything in life…there is a noticeable difference in the way that it grows…”*

(DG6).

While another observed that

*“… animals can produce better and be happier with human interaction”*

(DG3).

Here, participants not only acknowledged the intrinsic value of sentient farm animals but affirmed their extrinsic value: healthy and happy animals provide better quality produce.

As a condition of accepting the use of robots in agricultural contexts, several participants emphasised the importance of farm animals having the capacity to enjoy a natural life.

However, one dialogue group participant proposed a natural way of life for farm animals but run by AI and robots, inspired by their views on hunting wild animals:

*“… the only time they encounter a human is when they’re being shot. So I think that’s the best animal sometimes because it’s had the most free-range existence. But I think robots could be made to be less intimidating than humans. It doesn’t have to be this big scary machine…It could be how they’re fed with feeders and all that stuff. And if a chicken can go its life just chilling with other chickens and the AI makes its life better, then I’m all for it”*

(DG5).

Other participants reflected on how new technologies might support a natural life, and potentially offer a “symbiotic relationship between AI and humans” (DG6), for the benefit of animal welfare outcomes. Table 1 below highlights these three key themes that influence participant acceptance of robots in human–animal relationships in agriculture. The table provides insights from both the dialogue groups and community juries.

There were a small number of participants who professed to not having any real concern for farm animal welfare, unless, for instance, “… it’s too glaring, like it’s extremely bad…then it might become a concern” (DG7). As one participant explained, as a consumer, the main issues for them were cost, quality and availability of food. In other words, human interests trump animal welfare.

Finally, we also observed speciesism—a form of discrimination in which people assign more moral worth to one species (e.g., cows) over another species (e.g., chickens), evident in one farm worker’s comment that

*“… it’s harder for us to feel empathy with chooks…we’re more aware of the mammals that we keep, we’re more likely to think about that aspect”*

(DG1).

It is likely that some consumers may accept the use of robots in poultry—rather than other types of livestock production, given the different values assigned to different types of animal life.

## 5. Discussion

Our findings suggest that the removal of humans from all contact with farmed animals by highly automating animal agriculture would likely be rejected by the Australian public as being “a step too far”, largely due to its moral and social implications. However, should there be human oversight of robot interactions with farm animals, then hybrid (human–animal–robot) relations might well be accepted. Even so, public acceptance would depend on the context and conditions that help promote both human and animal wellbeing.

Overall, the scholarly literature focuses more on what is ethically permissible in the use of robots in animal agriculture, particularly as a framework for evaluating animal and human welfare. In contrast, our research examined the social acceptability of agricultural robots through the lens of the human–animal relationship. Our findings align with, and corroborate, the few studies on public perceptions of automation in animal agriculture that have been conducted. Specifically, that individuals

- Have concern for the loss of the human–animal relationship in farming [6,7];
- Place high value on the health and welfare of farm animals [6,34];
- Have misgivings about robots should the purpose be to replace farmers and intensify factory farming [7,34];
- Hold contrasting views, such as valuing modernity, innovation and progress while upholding a natural life for farm animals [6,34,36,43].

Below, we discuss our findings in more detail, weaving together the ethical literature with the intuitions of members of the Australian public. We do so, again, based on the three themes that underscored participant thinking around robots and the human–animal relationship in farming.

### 5.1. Moral Responsibility and Duty for Farm Animals

Some scholars have raised the question of whether autonomous robots should be considered moral agents and made responsible for their actions [44]. In the broader agricultural context, this becomes a question of who or what becomes responsible for decisions related to food production. While studies on agricultural robots have taken the potential implications of the use of robots for farmer decision-making seriously [45,46], the moral capabilities and decision-making abilities of robots are also relevant to the question of moral regard for nonhuman animals [47].

However, our participants largely rejected the idea that robots could assume responsibility for the lives of sentient farm animals. They questioned who would be responsible and accountable and in what ways. Members of the community juries argued that humans still owed animals a duty of care during slaughter. As one juror contended, even during slaughter, without human presence to minimise harms to sentient beings, it is the start of a “slippery slope” for animal welfare [10].

Regardless, most participants considered humans to hold moral responsibility for farm animals, insofar as they espoused that animal sentience has a moral standing. This fits with the philosopher Peter Singer’s claim that animal interests should be included in efforts to minimise pain and suffering and maximise pleasure, which guides human decision-making on how to act [48]. Participant views also suggest agreement with Singer and Tse (2023) that the interests of farm animals ethically matter [49].

### 5.2. Human Intuition and Empathy in Animal Care

Intensive animal agriculture arguably produces some of the most negative human–animal relations and involves significant abuse and neglect of animals by workers [19]. Participants in both the dialogue groups and the community juries acknowledged that preventing or minimising human cruelty toward farm animals might be a crucial benefit of agricultural robots. That said, they also held that completely automating animal agriculture would risk eliminating farmers’ tacit understanding in the care of farm animals. This unspoken “knowing” of an animal’s health and needs—gained from years of hands-on experience, forms a subjective relationship that participants believed technology cannot replicate. Participants described this relationship through the concept of the “human touch” or our ability to intuit or know how animals “feel”, which exemplifies tacit knowledge.

Their enthusiasm for the “human touch” supports Doidge and colleagues (2023), whose study highlights the farmer’s reliance on tacit knowledge or “having an eye for the animals” and reveals how farmer and animal bodies become interconnected through sensations which generate affective states [45]. Unlike automated farming practices, which objectify animals by understanding them only as data, “sensory knowledge symbolised by the farmers’ unity of hand, head and heart” [28], instead foregrounds the intrinsic value of an animal’s life. Such empathetic relations between farmers and the animals in their charge have long been associated with achieving good welfare outcomes for both [30].

Here, there was a productive analogy regarding care technologies in physician–patient–robot relations, raised by one medically trained participant. This participant suggested that AI is a useful tool for medical diagnosis and can potentially help reduce human error in high-stress, emotionally charged decision-making situations, However, this participant asserted that a doctor should still make the final decisions because individual anomalies and nuances impact diagnoses and treatment regimes. Understanding the nuances of the needs of individual patients requires human intuition, empathy and responsiveness [50,51]. In this regard, several of our participants would agree with those scholars who remain sceptical about the ability of robots to provide genuine care as, “… they cannot experience the emotions that are integral to the provision of such care” [52].

However, importantly, participants were not univocal in maintaining that human contact is necessarily beneficial for farmed animals. For example, a participant with farming experience described workers kicking or mistreating chickens, suggesting that robots might avoid this type of emotionally driven violence. This is an important observation because it complicates the association between human presence, empathy, and good animal welfare. The key question when it comes to the willingness of the public to accept the use of robots in agriculture, then, may be less about preserving human contact as such, and more about which forms benefit animals and which might usefully be reduced through automation. As mentioned earlier, the human–animal relationship in farming varies considerably across poultry and livestock systems and species [22]. Participant discussions, particularly within the dialogue groups, suggest that the implications of human empathy for animals may be different in relation to chickens and mammals. Their discussions also highlighted the ambiguity of care within animal agriculture for members of the public, which points to a tension: relations in care in farming coexist with relations of production, control, and slaughter. Hence, the acceptability of automation may be species and production system specific.

### 5.3. A Natural Life as a Condition of Animal Welfare

Western societies have, traditionally, approached agriculture through a human–animal distinction that places more value on human life than on its animal counterparts. Yet our inherent connectedness to nature is central to many indigenous epistemologies and cultures worldwide. While beliefs and understandings vary, these traditions tend to agree that all life is connected, alive, and possesses an identity or spirit [19]. Today, disciplines such as environmental ethics, ecological geography and the One Health and One Welfare approaches acknowledge the interconnectedness and interdependence of humans and nonhuman others [7,53,54].

Participants in our study were sympathetic towards the idea of an inherent human connectedness to nature. This view was particularly evident amongst those dialogue group participants who supported alternative farming practices that, in their view, allow animals to express their natural behaviours. Furthermore, this aligns with previous research indicating public support for the concept of naturalness in livestock production [6,35,55]. Similar to the findings of Vigors and colleagues (2021), while the perspectives of our dialogue group members were nuanced—balancing different aspects of animal welfare depending on the situation—they maintained that farm animals should have the opportunity for more natural lives by expressing natural behaviours akin to those of their wild counterparts [55]. We note that this suggests members of the public may prefer alternative systems, such as regenerative farming and silvopastoral systems, to intensive agriculture. For instance, a silvopastoral farm combines natural pasture and landscapes for livestock or poultry [56,57]. This approach provides animals with a rich environment, that improves their welfare, while offering farmers a way to maintain their traditional farming lifestyle [58]—an important consideration also raised by our dialogue group participants [9].

However, some of our participants also held that on-farm interactions between humans and animals were compatible with animals enjoying a natural life. A similar finding has previously been reported in a study by Bruckner and colleagues (2019), which showed that Austrian consumers and farmers involved in “alternative food networks” held that a natural life for farm animals does not necessarily equate to animals being left alone, but instead involves committed stewardship [59]. It remains possible, then, that at least some members of the public might endorse the use of robots as “helping hands” for care in the context of small-scale farming.

Finally, yet importantly, participants’ discussions of each theme raised key questions regarding the introduction of robots into animal agriculture, which we share in Table 2 below.

### 5.4. Study Limits and Strengths

Our previous studies examined public insights on the social and ethical implications of agricultural AI and robotics. In this companion paper, we have been specifically concerned with participant reflections on introducing robots into human–animal relationships in farming. Consequently, it is not exhaustive. Critical issues not examined here—such as full automation for biosecurity—raised broader welfare concerns regarding overcrowding, poor animal health, and devaluing of animal life, which we treated in a previous publication [9].

Variables such as previous experience with livestock, animal ownership, employment in agriculture, and urban versus rural background may have influenced participant perceptions, and analysis of their significance would have permitted more fine-grained findings. However, these variables were not recorded; consequently, we could not use these factors to interpret the findings more deeply or draw definitive conclusions.

In dialogue groups, the introduction of hypothetical scenarios can raise framing concerns. Our dialogue group participants did not necessarily have knowledge of automation in agriculture or current farming practices; hence, there was a risk that their attitudes could be shaped by the scenarios presented [9]. At times, these participants conflated “AI” with physical robots, suggesting their limited understanding of autonomous software and physical automation.

A community jury comprises small groups for high-quality deliberation; the downside is a resultant small sample size. There is potential for self-selection bias as each jury comprised participants largely interested in agriculture [10]. Having said that, both studies employed robust methodologies and provided fair representation of the views of members of the Australian public. In addition, the findings from both studies complemented one another, strengthening the overall credibility of our conclusions.

## 6. Concluding Remarks

Increasingly, people ask critical questions about production systems and expect attention be paid to their concerns around the welfare of farmed animals [43]. Involving the public in discourse on the future of robotics in animal agriculture may help keep an ethically fraught trajectory in check through their influence on upper-value-chain decision-making. Our findings suggest that key public concerns regarding the introduction of robots into the human–animal relationship in farming derive from deeper, underlying conceptions of animal sentience, intrinsic value, human caregiving, duties and responsibilities, and natural living; all of which form an ethical view of animal welfare [35].

Through our multi-structured engagement with members of the Australian public, we identified a set of conditions that citizens might expect to be met should robots (and other technologies) be introduced into the human–animal relationship in agriculture. These include the following:

- Human engagement and oversight to ensure respectful relations with (sentient) farm animals, and prevent the alienation of humans from nonhuman animals.
- Agricultural environments to provide conditions supporting natural behaviours and a near-to-natural life for farm animals, potentially supported by non-intrusive or animal-friendly robots.
- Technologies that support opportunities for maximising positive and minimising negative affective states in animals.
- Agricultural systems that support small-scale or family farms where robots work in partnership with humans.
- Clear lines of accountability, responsibility and integrity in animal farming.

Our study highlights a number of important ethical and policy questions that will arise should robots be introduced into the human–animal relationship in animal agriculture. Our research indicates that members of the Australian public value human oversight of (sentient) farmed animals in order to ensure animal welfare is sustained. The willingness of the public to accept agricultural robots into the human–animal relationship will depend on factors such as human moral responsibility for farm animals, the importance of empathy and tacit knowledge in their care, and a natural life as a condition of animal welfare. Public support for the advancement of robotic technologies in animal agriculture will likely be dependent upon meeting these and other ethical conditions and the impact of these technologies on the human–animal relationship.

## Figures and Tables

**Table 1:** Key themes around factors influencing participant acceptance of robots into the human–animal relationship in agriculture, across 12 dialogue groups and 2 community juries.

	12 Dialogue Groups (*n* = 67)	2 Community Juries (*n* = 23)
Themes	Group Insights	
**Sentience, moral responsibility & duty**	Farm animals have feelings; minimise harms, maximise pleasures. Human oversight of robots for animal welfare. Human duty of care; species needs vary. Humans accountable, not robots.	Human control to minimise harms to sentient beings. Human responsibility for animal lives. Levels of human accountability, not robots. A duty of care to animals during slaughter.
**Human intuition & empathy**	Robots cannot replicate human intuition, emotional understanding, empathy versus robots can simulate human care. Farmers intuit needs, understand nuances of individual animals, robots cannot. Robots potentially reduce harm from acts of human cruelty.	Animals react positively to human intuitive understanding & interactions. Should robots ‘take over’ there is potential for human detachment from “playing our part”.
**A natural life**	Chickens should lead a (near-to) natural life, able to express natural behaviours. Farmer as part of animals’ natural life versus natural life without humans. Robots risk loss of human connection with agriculture (as nature).	Humans as moral agents, aware of their relationship to the natural world & farming process of life & death.

**Table 2:** Key questions raised by dialogue group and community jury participants on introducing agricultural robots into the human–animal relationship.

Themes	Questions Raised
**Sentience, moral duty & responsibility**	Who’s responsible for the use and development of robots for animal agriculture and what will be their priorities? Does responsibility come down to an individual ‘calling the shots’, or humanity as a whole? Who’s policing? Who’s accountable? What would we be comfortable having no control over, and what would we require control over? Where is the guarantee that sentient beings are treated in a humane manner? Will there be transparency when it comes to how/where robots are used in animal agriculture?
**Human intuition & empathy**	On emotional intelligence and ethical treatment: Can machines detect specific animal behaviours? How can AI/robots intuit animal emotions like happiness, and to what extent?
**A natural life**	What are the effects on animals when they don’t have access to their natural environment? Can automation ‘look after’ animal welfare and the environment; is this achievable without humans?

## Data Availability

Data can be made available on reasonable request subject to restrictions imposed as part of ethics approval.
